# Supplementary material for: Structures and gating mechanisms of human bestrophin anion channels
Source: Nat Commun. 2022 Jul 4;13:3836. doi: 10.1038/s41467-022-31437-7 (PMC9253114; doi:10.1038/s41467-022-31437-7)
Supplement: Supplementary file 1 — Supplementary Information [file 41467_2022_31437_MOESM1_ESM.pdf]

Supplementary Information

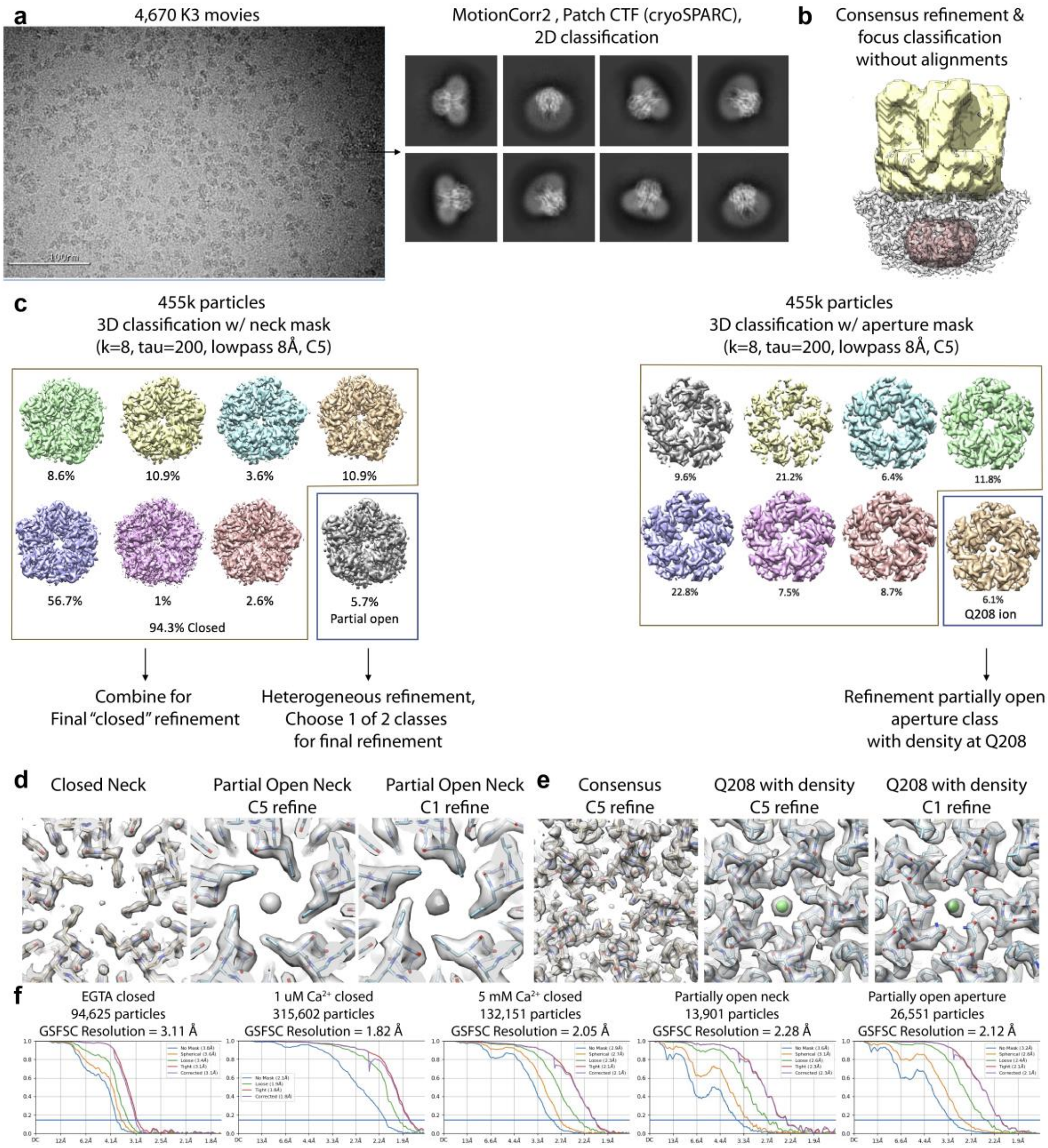

Supplementary Figure 1. Dataset characteristics and processing to identify the partially open state of hBest1. (**a**) Example aligned micrograph and 2D classes from cryoSPARC (2.12.4). (**b**) Consensus refinement with masks enclosing the TM domain (yellow) or aperture (red) used for focused 3D classification without alignments. (**c**) Particle separation scheme for 1 μM Ca^{2+} dataset revealing the partially open neck (left) and aperture (right), respectively. (**d**) Map and model for the closed neck (left column), partially open neck refined with C5 symmetry (middle column) and refined with no symmetry (right column) demonstrating the presence of an ion-like density bound at the edge of F84. (**e**) Map and Model for the aperture consensus refined with C5 symmetry (left column), partially open aperture refined with C5 symmetry (middle column) and refined with no symmetry (right column) demonstrating the presence of an ion-like density bound at the center of Q208. (**f**) FSC curves for indicated datasets.

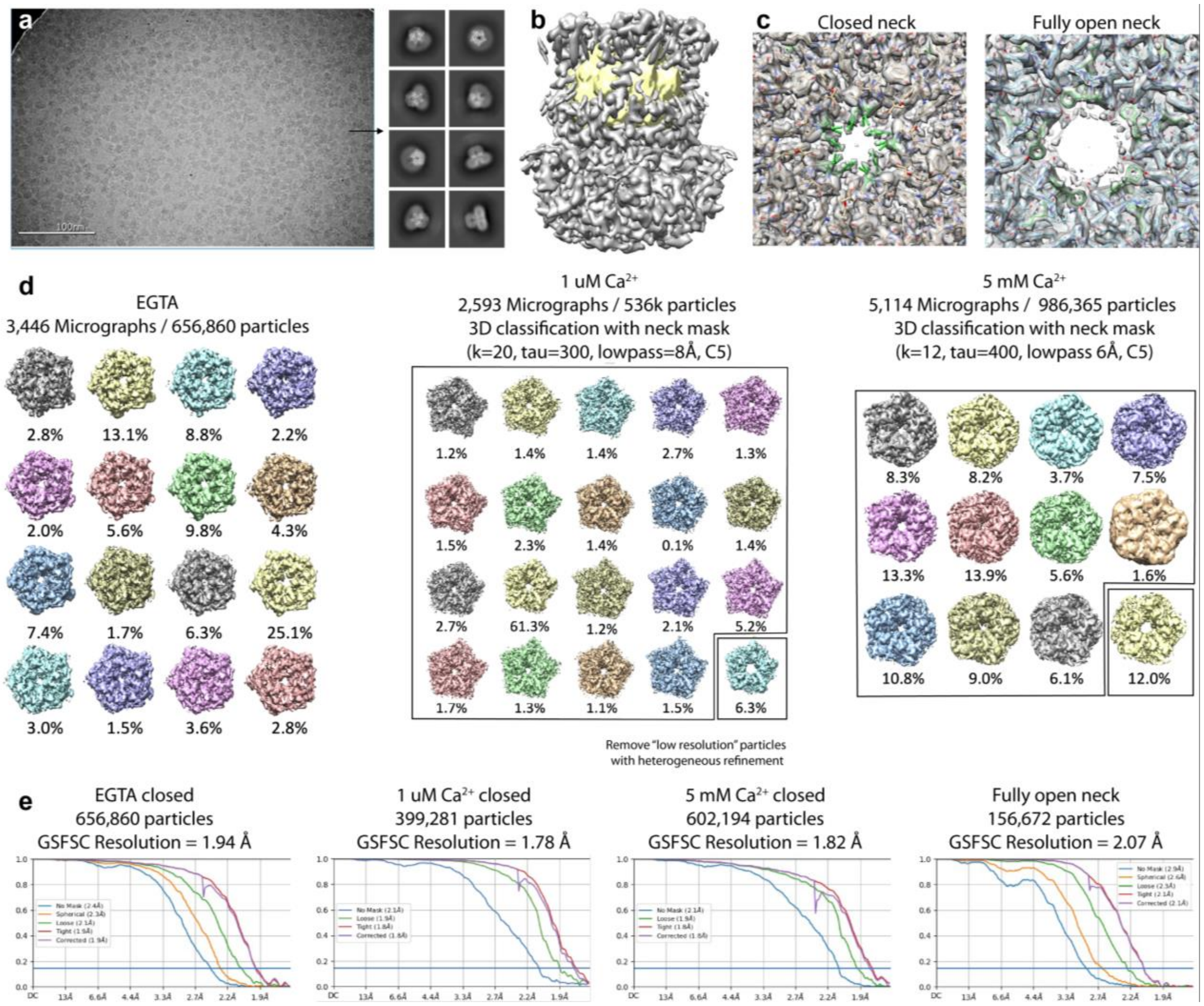

Supplementary Figure 2. Dataset characteristics and processing to identify the fully open state of hBest2. (**a**) Example aligned micrograph and 2D classes from cryoSPARC (2.12.4). (**b**) Consensus map with tight mask enclosing the neck-forming helices used for 3D classification. (**c**) *Left*, top view of map and model for the closed neck. *Right*, top view of map and model for the fully open neck. Neck-lining residues (I76, F80 and F84) shown as green sticks. (**d**) 3D classification scheme from each dataset. (**e**) FSC curves for indicated datasets.

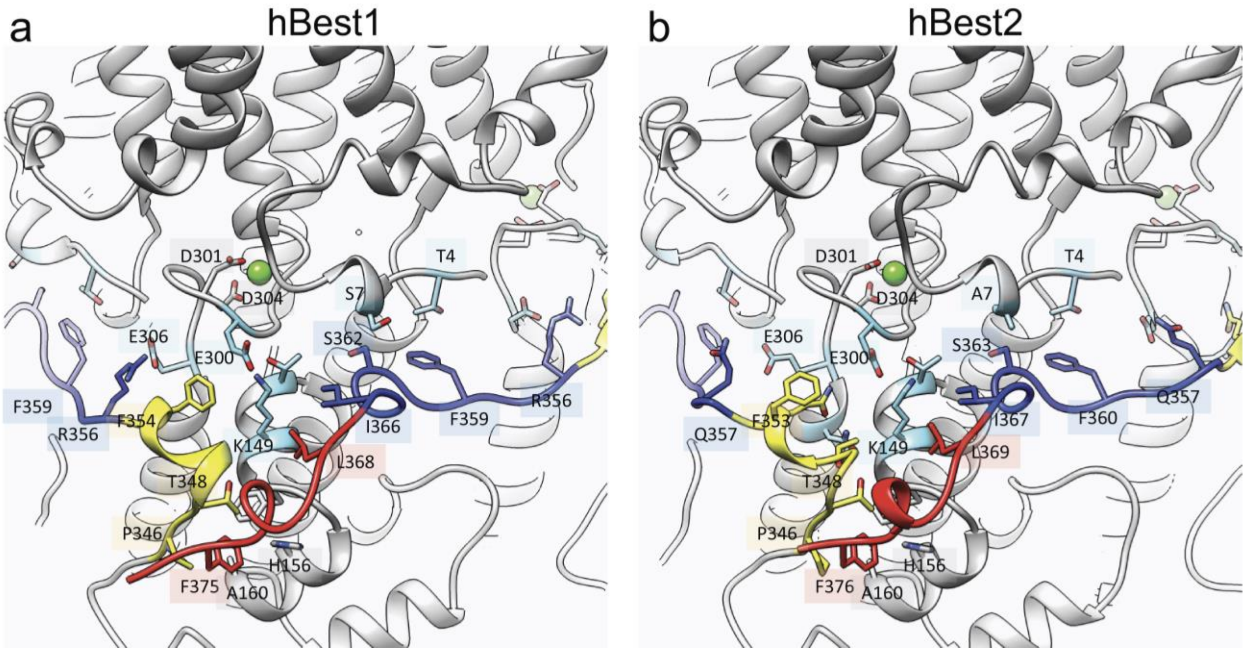

Supplementary Figure 3. The AS of the Ca^{2+}-bound closed state showing critical residues involved in channel gating. Residues making close contacts are shown as sticks for hBest1 (**a**) and hBest2 (**b**). The segments of the AS are colored as in Figure 3 (ACR1 in yellow, Anchor in blue, and ACR2 in red). Residues of the channel core that move in response to AS binding are shown as light blue sticks. Ca^{2+}-binding residues of the clasp are shown as gray sticks with Ca^{2+} ion shown as a green sphere. Note that the residue numbers of hBest1 and hBest2 after 353 are set off by one due to an insertion of a conserved F at position 353 in hBest2.

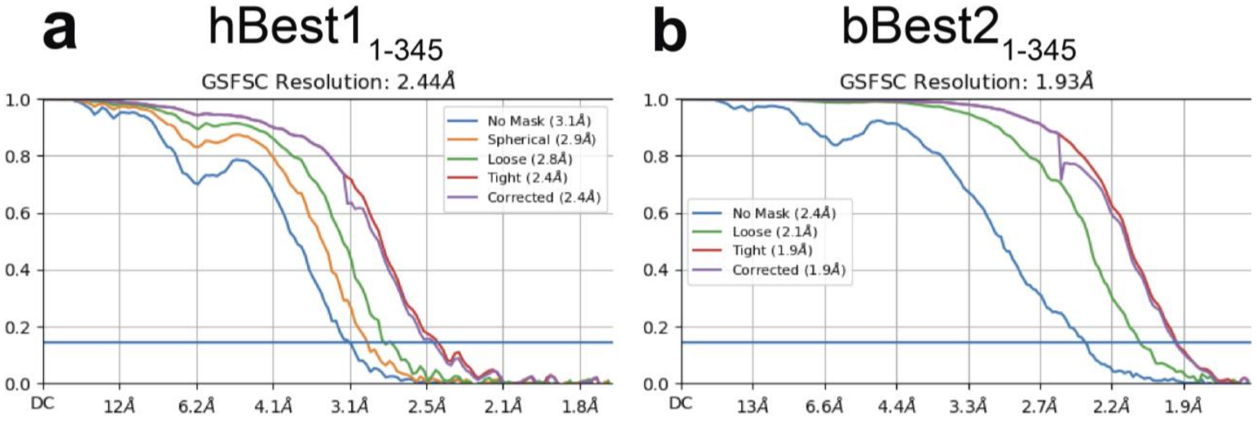

Supplementary Figure 4. Dataset characteristics and processing of hBest1$_{1\text{-}345}$ and bBest2$_{1\text{-}345}$. FSC curves for the cryo-EM maps from hBest1$_{1\text{-}345}$ (**a**) and bBest1$_{1\text{-}345}$ (**b**) datasets.

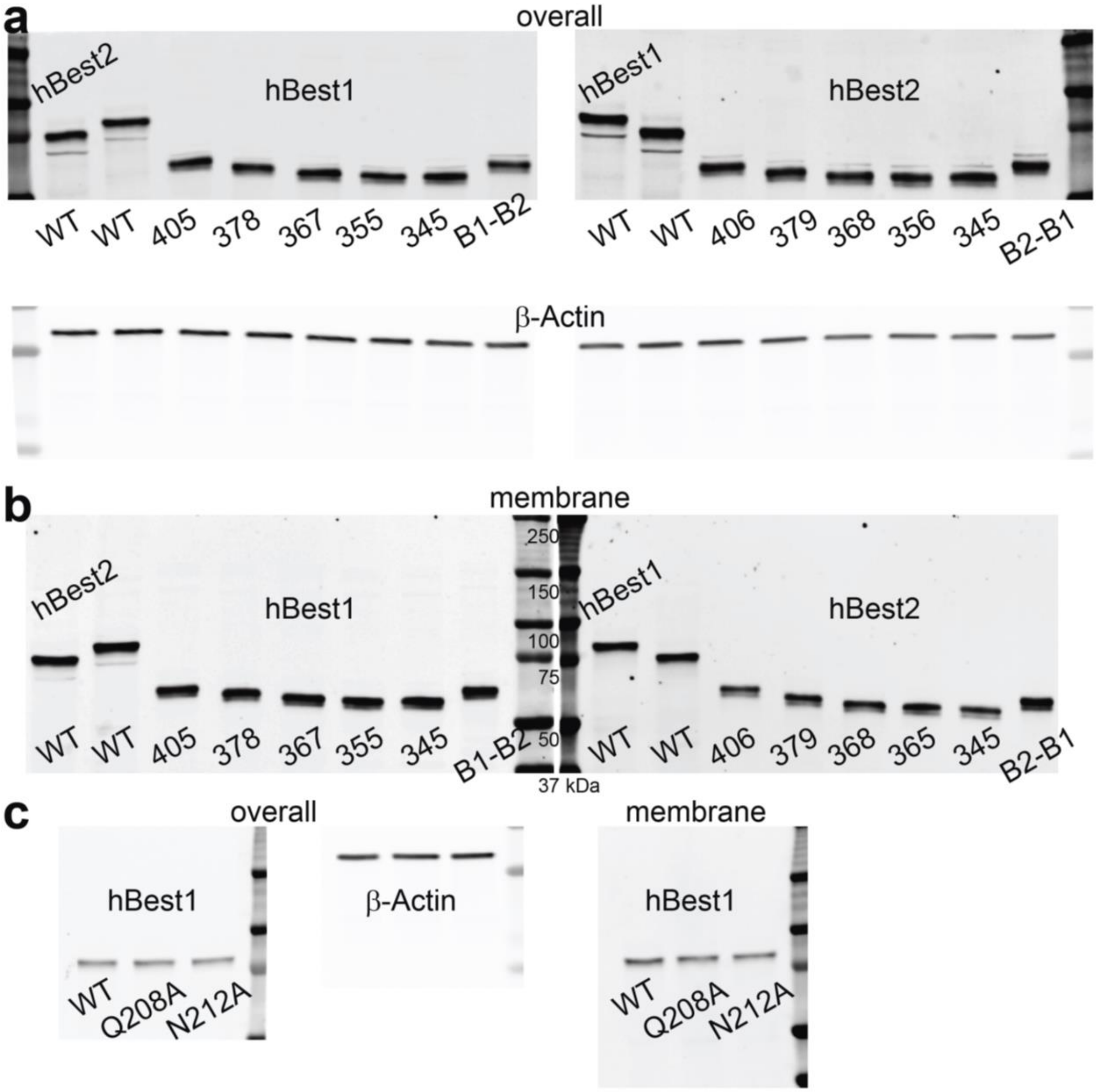

Supplementary Figure 5. Expression of hBest1 and hBest2 in transiently transfected HEK293 cells. (**a, b**) Representative blots showing the protein levels of full-length and truncated hBest1 and hBest2 in the total cell lysate (**a**) and crude membrane fraction (**b**) after transient transfection. B1-B2: hBest1$_{1\text{-}345}$-hBest2$_{346\text{-}406}$, B2-B1: hBest2$_{1\text{-}345}$-hBest1$_{346\text{-}405}$. (**c**) Representative blots showing the protein levels of WT and mutant hBest1 in the total cell lysate (left) and crude membrane (right) fraction after transient transfection. All hBest1 and hBest2 constructs were tagged with YFP and detected by an anti-GFP antibody. Each experiment was repeated three times independently with similar results.

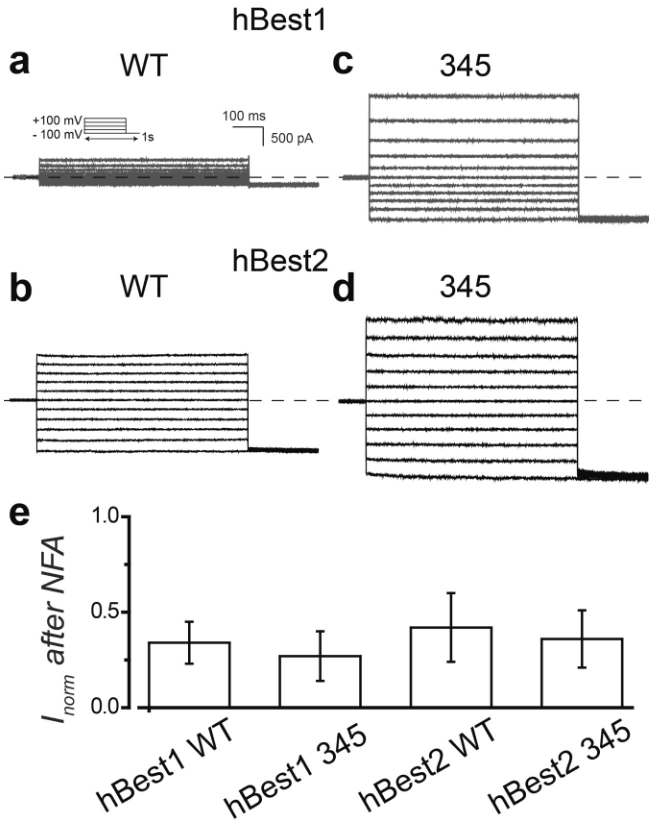

Supplementary Figure 6. Ca^{2+}-dependent Cl^- currents mediated by hBest1 and hBest2. (**a-d**) Representative current traces of WT hBest1 (**a**), WT hBest2 (**b**), $hBest1_{1\text{-}345}$ (**c**) and $hBest2_{1\text{-}345}$ (**d**) in transiently transfected HEK293 cells. Scale bar, 500 pA and 100 ms. Voltage protocol used to elicit currents is shown in *Inset*. (**e**) Normalized current densities from indicated constructs after 100 μM NFA treatment. n= 7-11 biologically independent cells for each bar. Data are presented as mean values +/- SEM. Source data and the precise n values are provided in the Source Data file.

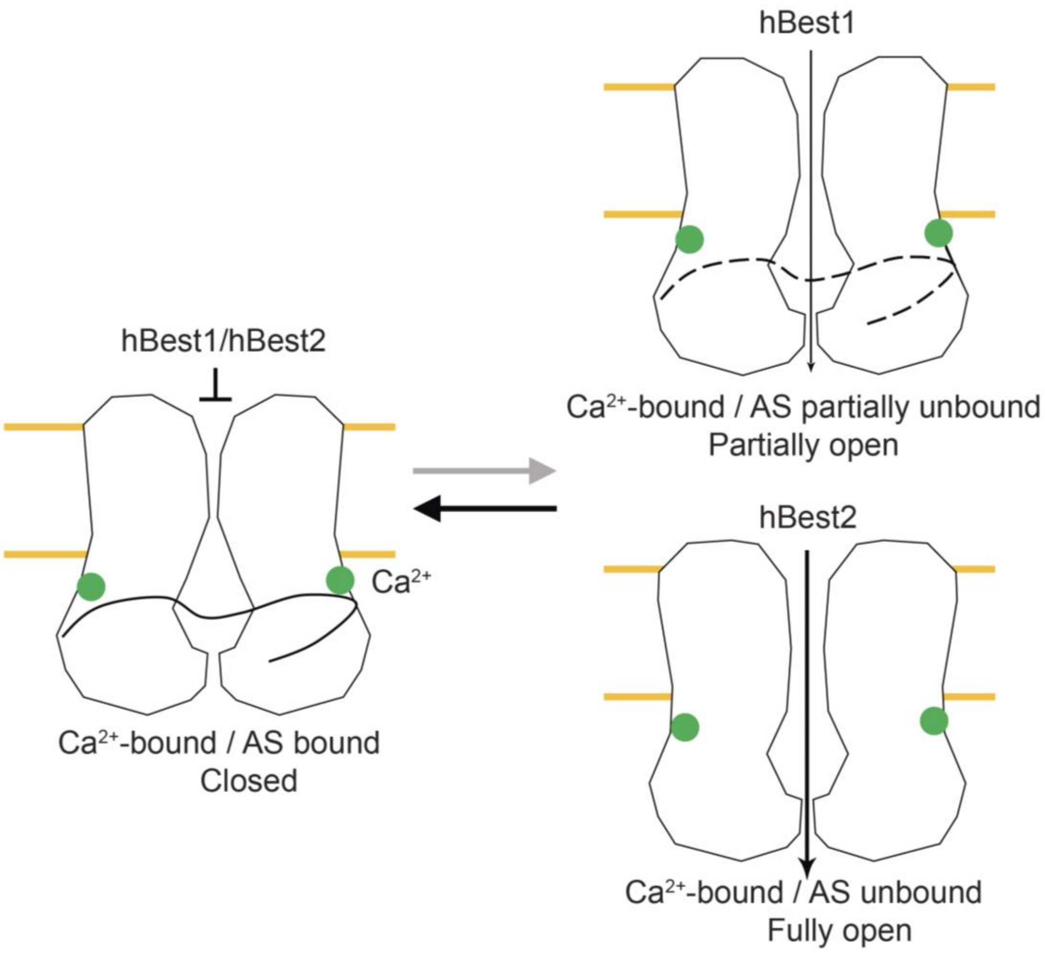

Supplementary Figure 7. A cartoon model showing the correlation between AS unbinding and neck opening in hBest1 and hBest2 channels.

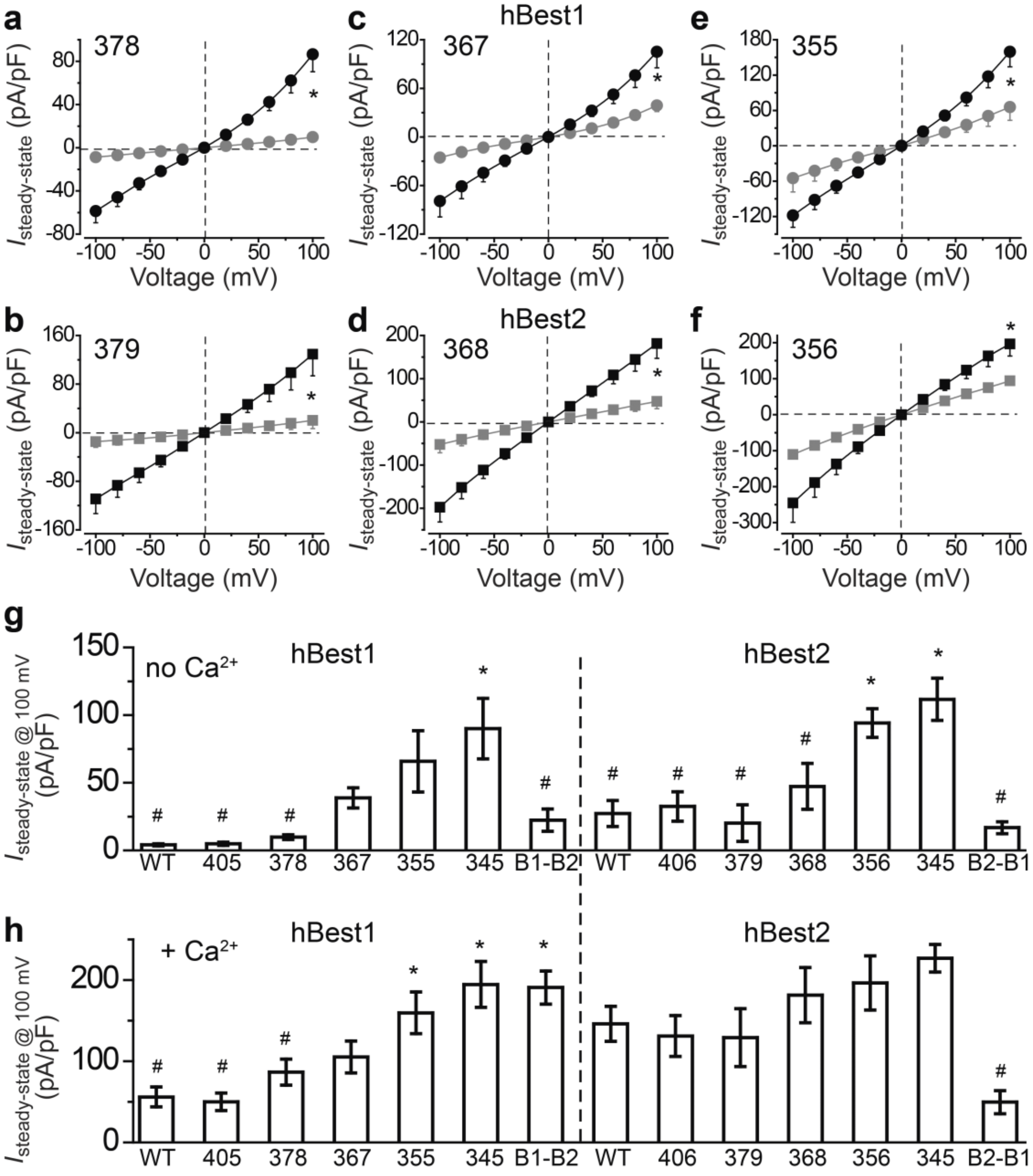

Supplementary Figure 8. Ca^{2+}-dependent Cl^- currents mediated by hBest1 and hBest2. (**a**-**f**) Population steady-state current density-voltage relationships in the absence (gray) and presence (black) of 1 μM Ca^{2+} from HEK293 cells expressing hBest$1_{1\text{-}378}$ (**a**), hBest$1_{1\text{-}367}$ (**c**), hBest$1_{1\text{-}355}$ (**e**), hBest$2_{1\text{-}379}$ (**b**), hBest$2_{1\text{-}368}$ (**d**), or hBest$2_{1\text{-}356}$ (**f**); n= 5-14 biologically independent cells for each point. *P < 0.05 compared to currents without Ca^{2+}, using two-tailed unpaired Student t test. (**g**, **h**) Bar chart showing the steady-state current densities from HEK293 cells expressing the indicated channels in the absence (**g**) and presence (**h**) of 1 μM Ca^{2+}, n= 5-15 biologically independent cells for each bar. *#Significantly different (P < 0.05) compared to currents from WT and 1-345 under the same condition, respectively, using one-way ANOVA followed by Bonferroni *post hoc* means comparisons. B1-B2, hBest$1_{1\text{-}345}$-hBest$2_{346\text{-}406}$; B2-B1, hBest$2_{1\text{-}345}$-hBest$1_{346\text{-}405}$. Data are presented as mean values +/- SEM. Source data and the precise n and P values are provided in the Source Data file.

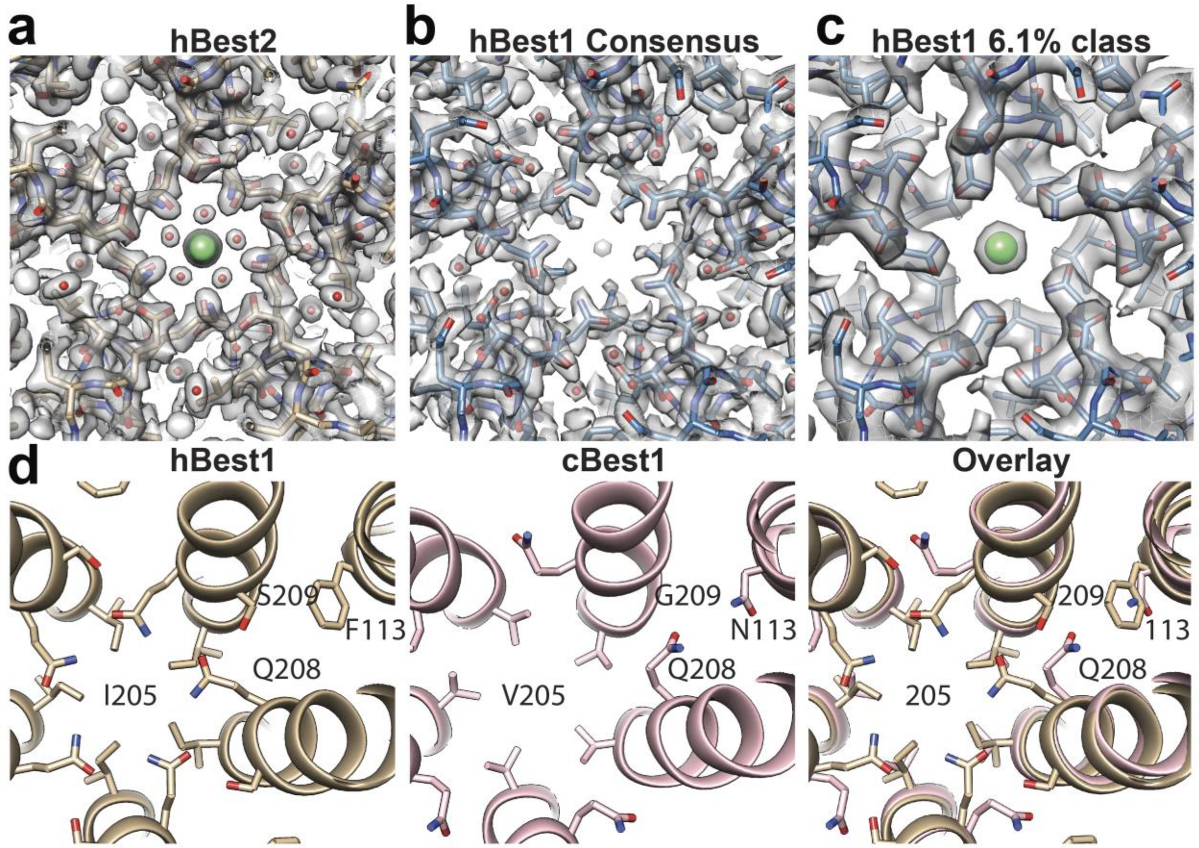

Supplementary Figure 9. Comparison of the bestrophin apertures. (**a-c**) Map and model of the aperture, viewed from within the cytosolic vestibule, for hBest2 (**a**), hBest1 consensus (**b**), and hBest1 rare class with density at Q208 (**c**). (**d**) Aperture of hBest1 (tan) and cBest2 (pink) showing critical residues involved in maintaining Q208 conformation. S209 makes a hydrogen bond with Q208 to coordinate the side chain in hBest1, while the analogous G209 in cBest1 allows Q208 to take a different conformation away from the pore.

Supplementary Table 1. cryo-EM data collection and refinement statistics

State	hBest1_1uM closed	hBest1_5mM closed	hBest1_1uM neck partially open	hBest1_1uM aperture partially open	hBest1_EGTA closed	hBest1_345 open
PDB	8D1I	8D1J	8D1K	8D1L	8D1M	8D1O
EMDB	27131	27132	27133	27134	27135	27137
Data collection and processing						
Microscope	Columbia Titan Krios	Columbia Titan Krios	Columbia Titan Krios	Columbia Titan Krios	Columbia Titan Krios	Columbia Titan Krios
Detector	K3	K3	K3	K3	K3	K3
Magnification	105,000	105,000	105,000	105,000	105,000	105,000
Voltage (kV)	300	300	300	300	300	300
Electron Exposure (e^-/Å^2)	58	58	58	58	58	58
Defocus Range (μm)	1.0 - 2.0	1.0 - 2.0	1.0 - 2.0	1.0 - 2.0	1.0 - 2.0	1.0 - 2.0
Pixel Size	0.83	0.83	0.83	0.83	0.83	0.83
Final symmetry imposed	C5	C5	C5	C5	C5	C5
Micrographs (#)	4,670	2,047	4,670	4,670	5,558	2,138
Initial particle images (#)	1,727,992	752,738	1,727,992	1,727,992	2,079,411	803,036
Final particle images (#)	315,602	132,151	13,901	26,551	94,625	25,050
Map resolution (Å)	1.82	2.05	2.28	2.12	3.11	2.44
FSC threshold	0.143	0.143	0.143	0.143	0.143	0.143
Refinement						
Initial models used	hBest1_5mM	Swissmodel from 6vx7	hBest1_5mM	hBest1_5mM	hBest1_5mM	hBest1_5mM
Model composition						
Non-hydrogen atoms	17,175	17,151	16,305	16,306	15,590	15,015
_Protein residues	1,880	1,880	1,880	1,880	1,880	1,690
_Ligand	5 Ca^{2+} / 40 MC3	5 Ca^{2+} / 40 MC3	5 Ca^{2+} / 40 MC3	5 Ca^{2+} / 1 Cl^- / 40 MC3	-	5 Ca^{2+} / 40 MC3
B factor (mean, Å2)						
_Protein	45.04	12.71	45.89	46.97	55.33	76.38
_Ligand	132.67	28.43	6.59	60.33	-	139.27
R.m.s. deviations						

_Bond lengths (Å) (# > 4 sigma)	0.012 (0)	0.004 (0)	0.013 (0)	0.004 (0)	0.006 (0)	0.015 (0)
_Bond angles (deg.) (# > 4 sigma)	1.423 (5)	0.651 (15)	1.528 (0)	0.612 (5)	0.865 (5)	1.836 (0)
Validation						
_MolProbity score	1.39	1.42	1.68	1.25	1.83	1.67
_Clashscore	7.11	7.23	6.44	4.07	12.63	11.98
_Poor rotamers (%)	0.94	1.06	2.65	1.18	1.18	0.33
Ramachandran Plot						
_Favored (%)	99.36	98.93	98.93	98.4	97.06	97.62
_Allowed (%)	0.64	1.07	1.07	1.6	2.67	2.38
_Disallowed (%)	0	0	0	0	0.27	0
EMRinger Score	7.49	5.87	4.11	4.78	3.5	4.65

State	hBest2_1uM closed	hBest2_5mM closed	hBest2 open	hBest2_EGTA closed	bBest2_345 open
PDB	8D1E	8D1F	8D1G	8D1H	8D1N
EMDB	27127	27128	27129	27130	27136
Data collection and processing					
Microscope	Columbia Titan Krios	Columbia Titan Krios	Columbia Titan Krios	Columbia Titan Krios	Columbia Titan Krios
Detector	K3	K3	K3	K3	K3
Magnification	105,000	105,000	105,000	105,000	105,000
Voltage (kV)	300	300	300	300	300
Electron Exposure ($e^-/Å^2$)	58	58	58	58	58
Defocus Range (μm)	1.0 - 2.0	1.0 - 2.0	1.0 - 2.0	1.0 - 2.0	1.0 - 2.0
Pixel Size	0.83	0.83	0.83	0.83	0.83
Final symmetry imposed	C5	C5	C5	C5	C5
Micrographs (#)	2,593	5,114	7,707	3,446	3,203
Initial particle images (#)	2,040,714	3,419,200	3,419,200	2,853,381	1,070,653
Final particle images (#)	399,281	602,194	156,672	656,860	117,555
Map resolution (Å)	1.78	1.82	2.07	1.94	1.93

FSC threshold	0.143	0.143	0.143	0.143	0.143
Refinement					
Initial models used (PDBid)	Swissmodel from 6vx7	hBest2_1uM	hBest2_1uM	hBest2_1uM	6vx7
Model composition					
Non-hydrogen atoms	17,340	17,376	16,201	17,156	15,836
_Protein residues	1,880	1,880	1,735	1,880	1,690
_Ligand	5 Ca^{2+} / 1 Cl^{-} / 5 DU0 / 50 MC3	5 Ca^{2+} / 1 Cl^{-} / 5 DU0 / 50 MC3	5 Ca^{2+} / 1 Cl^{-} / 5 DU0 / 50 MC3	1 Cl^{-} / 5 DU0 / 45 MC3	5 Ca^{2+} / 1 Cl^{-} / 5 DU0 / 50 MC3
B factor (mean, Å2)					
_Protein	45.61	9.15	20.5	10.58	4.25
_Ligand	125.09	22.35	125	36.41	14.84
R.m.s. deviations					
_Bond lengths (Å) (# > 4 sigma)	0.012 (0)	0.006 (0)	0.007 (0)	0.008 (0)	0.009 (0)
_Bond angles (deg.) (# > 4 sigma)	1.332 (0)	1.329 (19)	1.007 (0)	1.112 (0)	1.408 (0)
Validation					
_MolProbity score	1.37	1.32	1.64	2.09	1.6
_Clashscore	6.73	5.79	13.54	16.2	12.14
_Poor rotamers (%)	0.61	0.73	0.66	2.13	0
Ramachandran Plot					
_Favored (%)	98.4	98.66	98.83	97.33	98.81
_Allowed (%)	1.6	1.34	1.17	2.67	1.19
_Disallowed (%)	0	0	0	0	0
EMRinger Score	7.64	7.49	5.81	6.44	7.81

Supplementary Table 2. Sequences of oligonucleotides

Primer name	Sequence
hBest1-F	TCACTAGTCGCGGCCGCATGACCATCACTTACACAAGCCA
hBest1-345-R	GAAGTAGAGGTTCTCGGGCTGTGGCTCGGG
hBest1-355-R	GAAGTAGAGGTTCTCACGGAACTGGGCGGAA
hBest1-367-R	GAAGTAGAGGTTCTCGCTGATGTTGAAGGTGGAGCC
hBest1-378-R	GAAGTAGAGGTTCTCATTGGGCTGGAACTCCATCTC
hBest1-405-R	GAAGTAGAGGTTCTCCCTGGGAGGATGGTGATCAT
hBest1-FL-R	GAAGTAGAGGTTCTCGGAATGTGCTTCATCCCTGTT
hBest1-345-hBest2-406-F	CCATACACAGCGGCTACTGT
hBest1-345-hBest2-406-R	AGCCGCTGTGTATGGGGGCTGTGGCTC
hBest2-F	TCACTAGTCGCGGCCGCATGACCGTCACCTACACAGCC
hBest2-345-R	GAAGTAGAGGTTCTCGGCGCGAGCCTCG
hBest2-356-R	GAAGTAGAGGTTCTCCCGCAGCTGGAAGACAGTA
hBest2-368-R	GAAGTAGAGGTTCTCCGTGATGTCAAAGGTGGAGC
hBest2-379-R	GAAGTAGAGGTTCTCCAGCCGCTGGAACTGCAT
hBest2-406-R	GAAGTAGAGGTTCTCCGCGACCATGCCCG
hBest2-FL-R	GAAGTAGAGGTTCTCGGCCAGATTCTCCTCCTCCT
hBest2-345-hBest1-405-F	CCCTACACAGCTGCTTCC
hBest2-345-hBest1-405-R	AGCAGCTGTGTAGGGGGCGCGAGCC
