# Supplementary material for: Structures and gating mechanisms of human bestrophin anion channels
Source: Nat Commun. 2022 Jul 4;13:3836. doi: 10.1038/s41467-022-31437-7 (PMC9253114; doi:10.1038/s41467-022-31437-7)
Supplement: Supplementary file 3 — Description of Additional Supplementary Files [file 41467_2022_31437_MOESM3_ESM.pdf]

Description of Additional Supplementary Files

File Name: Supplementary Movie 1
Description: **3DVA movies for Ca^{2+}-bound hBest2.** Coordinated ordering and disordering of the AS between the Ca^{2+}- bound closed and open states. Frames of 3DVA for hBests2 from side and top views were visualized as a volume series. Residues of the neck are colored pink and the regions of the AS are colored in the same manner as in Figure 3 (ACR1 in yellow, Anchor in blue, and ACR2 in red).

File Name: Supplementary Movie 2
Description: **3DVA movies for Ca^{2+}-bound hBest1.** Coordinated ordering and disordering of the AS between the Ca^{2+}- bound closed and open states. Frames of 3DVA for hBest1 from side and top views were visualized as a volume series. Residues of the neck are colored pink and the regions of the AS are colored in the same manner as in Figure 3 (ACR1 in yellow, Anchor in blue, and ACR2 in red).
