# Supplementary material for: Structures and gating mechanisms of human bestrophin anion channels
Source: Nat Commun. 2022 Jul 4;13:3836. doi: 10.1038/s41467-022-31437-7 (PMC9253114; doi:10.1038/s41467-022-31437-7)
Supplement: Supplementary file 6 — Reporting Summary [file 41467_2022_31437_MOESM6_ESM.pdf]

nature research

Corresponding author(s): Tingting Yang

Last updated by author(s): May 31, 2022

Reporting Summary

Nature Research wishes to improve the reproducibility of the work that we publish. This form provides structure for consistency and transparency in reporting. For further information on Nature Research policies, see Authors & Referees and the Editorial Policy Checklist .

Statistics

For all statistical analyses, confirm that the following items are present in the figure legend, table legend, main text, or Methods section.

n/a	Confirmed	
☐	☒	The exact sample size (n) for each experimental group/condition, given as a discrete number and unit of measurement
☐	☒	A statement on whether measurements were taken from distinct samples or whether the same sample was measured repeatedly
☐	☒	The statistical test(s) used AND whether they are one- or two-sided<br>*Only common tests should be described solely by name; describe more complex techniques in the Methods section.*
☒	☐	A description of all covariates tested
☒	☐	A description of any assumptions or corrections, such as tests of normality and adjustment for multiple comparisons
☐	☒	A full description of the statistical parameters including central tendency (e.g. means) or other basic estimates (e.g. regression coefficient) AND variation (e.g. standard deviation) or associated estimates of uncertainty (e.g. confidence intervals)
☐	☒	For null hypothesis testing, the test statistic (e.g. F, t, r) with confidence intervals, effect sizes, degrees of freedom and P value noted<br>*Give P values as exact values whenever suitable.*
☒	☐	For Bayesian analysis, information on the choice of priors and Markov chain Monte Carlo settings
☒	☐	For hierarchical and complex designs, identification of the appropriate level for tests and full reporting of outcomes
☒	☐	Estimates of effect sizes (e.g. Cohen's d, Pearson's r), indicating how they were calculated

Our web collection on statistics for biologists contains articles on many of the points above.

Software and code

Policy information about availability of computer code

Data collection	Patchmaster v2x90.5, Leginon, Appion
Data analysis	Patchmaster v2x90.5, Origin, Motioncor2, Relion 3.0, cryoSPARC, pyem, Phenix, Coot, Molprobity, SWISS-MODEL, Chimera, HOLE, Servalcat, REFMAC5, EMRinger

For manuscripts utilizing custom algorithms or software that are central to the research but not yet described in published literature, software must be made available to editors/reviewers. We strongly encourage code deposition in a community repository (e.g. GitHub). See the Nature Research guidelines for submitting code & software for further information.

Data

Policy information about availability of data

All manuscripts must include a data availability statement. This statement should provide the following information, where applicable:

- Accession codes, unique identifiers, or web links for publicly available datasets
- A list of figures that have associated raw data
- A description of any restrictions on data availability

All relevant data are available from the corresponding author upon reasonable request. Source data are provided in the supplementary information file. The structures in this paper are deposited to the Protein Data Bank and Electron Microscopy Data Bank with the following access codes: 8D1E, EMD-27127; 8D1F, EMD-27128; 8D1G, EMD-27129; 8D1H, EMD-27130; 8D1I, EMD-27131; 8D1J, EMD-27132; 8D1K, EMD-27133; 8D1L, EMD-27134; 8D1M, EMD-27135; 8D1N, EMD-27136; 8D1O, EMD-27137.

Field-specific reporting

Please select the one below that is the best fit for your research. If you are not sure, read the appropriate sections before making your selection.

[x] Life sciences [ ] Behavioural & social sciences [ ] Ecological, evolutionary & environmental sciences

For a reference copy of the document with all sections, see nature.com/documents/nr-reporting-summary-flat.pdf

Life sciences study design

All studies must disclose on these points even when the disclosure is negative.

Sample size	Sample sizes were indicated in the legend of each Figure and Supplementary Figure. No statistical methods were used to predetermine sample sizes. We determined sample sizes as follows: for immunoblotting experiments, we performed triplicate experiments and for patch clamp recordings at least n=5 for each group, to meet the minimal requirements for statistical analysis.
Data exclusions	No data were excluded.
Replication	All experiments were replicated for at least three times. All attempts at replications were successful.
Randomization	cells were randomly assigned to the experimental groups.
Blinding	Investigators were blinded to groups allocation during data collection.

Reporting for specific materials, systems and methods

We require information from authors about some types of materials, experimental systems and methods used in many studies. Here, indicate whether each material, system or method listed is relevant to your study. If you are not sure if a list item applies to your research, read the appropriate section before selecting a response.

Materials & experimental systems

n/a	Involved in the study	
[ ]	[x]	Antibodies
[ ]	[x]	Eukaryotic cell lines
[x]	[ ]	Palaeontology
[x]	[ ]	Animals and other organisms
[x]	[ ]	Human research participants
[x]	[ ]	Clinical data

Methods

n/a	Involved in the study	
[x]	[ ]	ChIP-seq
[x]	[ ]	Flow cytometry
[x]	[ ]	MRI-based neuroimaging

Antibodies

Antibodies used	rabbit anti-GFP (Thermo Fisher Scientific, A-6455), mouse anti-β-Actin (Thermo Fisher Scientific, MA5-15739), IRDye® 680RD goat anti-rabbit IgG (LI-COR, 926-68071), IRDye® 680RD goat anti-mouse IgG (LI-COR, 926-68070)
Validation	rabbit anti-GFP, RRID:AB_221570<br>mouse anti-β-Actin, RRID:AB_10979409<br>IRDye® 680RD goat anti-rabbit IgG, RRID AB_10956166<br>IRDye® 680RD goat anti-mouse IgG, RRID AB_10956588

Eukaryotic cell lines

Policy information about cell lines

Cell line source(s)	HEK293 and HEK293-F cells were kindly gifted from Drs. Henry Colecraft and Ravi Kalathur, respectively. HEK293 cells were originally purchased from ATCC. HEK293-F cells were originally purchased from ThermoFisher.
Authentication	Cells were authenticated by short tandem repeat DNA profiling. RRID:CVCL_0045, RRID:CVCL_6642
Mycoplasma contamination	No mycoplasma contamination was found.
Commonly misidentified lines (See ICLAC register)	HEK293.
